# Simple neutralization test report: Do probiotics contribute to COVID-19 therapy?

**DOI:** 10.1016/j.bbrep.2022.101348

**Published:** 2022-09-13

**Authors:** Anna Surgean Veterini, Cita Rosita Sigit Prakoeswa, Damayanti Tinduh, Satuman Satuman

**Affiliations:** a Anesthesiology and Reanimation Department, Faculty of Medicine, Universitas Airlangga - Dr Soetomo General Academic Hospital, Surabaya, 60286, Indonesia; b Dermatology Department, Faculty of Medicine, Universitas Airlangga - Dr Soetomo General Academic Hospital, Surabaya, 60286, Indonesia; c Medic Rehabilition Department, Faculty of Medicine, Universitas Airlangga - Dr Soetomo General Academic Hospital, Surabaya, 60286, Indonesia; d Physiology Department, Faculty of Medicine, Universitas Brawijaya, Malang, 65145, Indonesia

**Keywords:** Neutralisation test, Protein spikes recombinant, *in vitro* COVID-19 models, Probiotics for COVID-19, IgG sRBD

## Abstract

**Background:**

There is an urgent need to identify effective therapy to treat coronavirus diseases 2019 (COVID-19). Supplement consumption is becoming popular in this pandemic era. An example of this is probiotic consumption to improve the host's immune system.

**Objective:**

This study aimed to prove whether antibodies from people taking probiotics could influence lactate dehydrogenase (LDH), adenosine triphosphate (ATP) values, and cell viability *in vitro* in peripheral blood mononuclear cells (PBMCs) inoculated with the SARS-CoV-2 spike protein as COVID-19 cells models.

**Methods:**

This was an experimental study with control and intervention groups, totally in 12 groups divided based on antibody levels, probiotic intervention, probiotic non-intervention group, SARS-CoV-2 infection group, and non-SARS-CoV-2 infection group. *In vitro* assays were carried out on PBMC cell cultures inoculated with S1 SARS-CoV-2 recombinant as a COVID-19 cell model. The COVID-19 cell model was given antibodies divided into three antibody level groups: sRBD levels of <3, 325.76 and 646.18. The cytotoxicity assessment examined increased levels of LDH, cytopathic activity by measuring ATP levels, and cell viability by XTT (2,3-Bis-(2-Methoxy-4-Nitro-5-Sulfophenyl)-2*H*-Tetrazolium-5-Carboxanilide) assay. Data were analyzed with SPSS 21 for Windows.

**Results:**

This study showed a significant difference in the LDH value (p < 0.001) between each group. The difference in ATP values between groups was significant (p < 0.001). Meanwhile, the cell viability examination found that there was a tendency of decreased XTT (cell viability in %) when there was an increase of LDH and ATP.

**Conclusion:**

The change of LDH values occurred most in the antibody group that did not consume probiotics. The highest cytopathic activity based on the ATP values occurred in the infected cell culture group with antibody levels of 325.76 and consuming probiotics. In addition, LDH and ATP activities provided evidence of a significant influence on cell viability.

## Highlights

• Antibody from subject who consumed probiotic perform higher values in cell viability assay (XTT).
• Higher antibody titer (IgG) didn't determine better performance in neutralisation test.
• There is a tendency to decrease XTT if the ATP and LDH values are increased.

## 1 Introduction

The global COVID-19 pandemic has prompted experts to continue researching the most effective therapy for the virus. Up to date, there has been no estabished medication to eradicate the SARS-CoV-2 virus and to achieve the desired outcomes. The nature and duration of protective immunity are crucial factors to consider when assessing the risk of reinfection and developing vaccines [1,2].

A discourse written by Bozkurt and Quigley (2020) states that probiotics are living microorganisms that have been shown to have positive effects on immune responses in human. Some strains of *Bifidobacteria,* for example, possess incredibly potent immune-modulating effects. These bacteria have the potential to ameliorate the ‘cytokine storm’ through a differential impact on pro- and anti-inflammatory cytokines. Probiotic bacteria can also enhance vaccine efficacy in managing COVID-19 and other coronavirus-mediated illnesses [3].

In the previous literature, it has been proven by Pormoontaseri (2017) *in vitro* that probiotics can inhibit cytotoxic effects due to *Clostridium perfringens* infection and may promote better cell viability [4,5]. Unfortunately, the molecular basis on how probiotics demonstrate therapeutic effects in preventing viral infections remains unclear. In COVID-19 patients, an apparent drop of *Lactobacillus* and *Bifidobacterium* sp. Cell counts, both of which are well-known providers of gut probiotics, has been found [6].

According to studies by Banerjee et al., probiotics can reduce the cytotoxic and cytopathic consequences of viral infections [7]. Probiotics work by directly interacting with viruses and stimulating the immune system to prevent viral infection [8].

*In vitro* models of host-pathogen interactions are critical for understanding the infectious disease process and assessing antimicrobial drug efficiency. The lactate dehydrogenase (LDH) cytotoxicity assay and the ATP cytopathic assay were used to assess the viability of the PBMC-induced spike protein SARS-CoV-2 in these models.

Neutralisation tests with serum antibodies in cases of SARS-CoV-2 infection may be difficult and dangerous because they must be carried out in a biosafety level 3 (BSL-3) laboratory. A method to overcome this is to use an S1 SARS COV-2 protein recombinant. This study tested the serum antibody neutralisation of COVID-19 survivors who consumed probiotics and those who did not through *in vitro* research on COVID-19 model cells.

## 2 Methods

This study has been granted ethical permission from the Ethical Committee of Dr. Soetomo General Academic Hospital, with the ethical conduct number: 0117/LOE/301.4.2/IX/2020. The study was conducted at RSUD Dr. Soetomo Surabaya and Universitas Brawijaya Malang.

### 2.1 IgG sRBD analysis

Thirty subjects were patients with COVID-19 and were divided into 2 groups: groups that consumed probiotics (15 subjects) and did not consume probiotics (15 subjects). All subjects consumed probiotics starting from being confirmed as COVID-19 until taking blood samples for sRBD examination. They consumed probiotics for 30 days on average. The serum IgG sRBD antibody levels were measured by prospectively collecting blood samples from subjects on the 21st day after being declared negative for SARS-COV-2 infection. The measurement was performed using the chemiluminescence immunoassay (CLIA) reagent kit manufactured by Shenzen Mindray Bio-Medical Electronic CO., LTD. Probiotics used in this study were containing *Lactobacillus plantarum* EMRO 009 (2.0 × 10^6^ cfu/ml)*, Lactobacillus casei* EMRO 002 (2.0 × 10^6^ cfu/ml)*, Lactobacillus casei* EMRO 213 (2.0 × 10^6^ cfu/ml)*, Lactobacillus fermentum* EMRO 211 (2.0 × 10^6^ cfu/ml)*, Lactobacillus bulgaricus* EMRO 212 (2.0 × 10^6^ cfu/ml)*, Lactobaccilus rhamnosus* EMRO 014 (2.0 × 10^6^ cfu/ml)*,* and *Rhodopseudomonas palustris* EMRO 201 (>2.0 × 10^6^ cfu/ml, honey (0.17 ml), aloe vera juice (0.16 ml)*.* Subjects consumed probiotics by oral 15 ml three times a day for at least 1 month. All the subjects in these two groups of this study received anti-viral therapy, vitamin C and vitamin D which were the standard therapy from the hospital. We gave probiotics as adjunct therapy in the intervention group. The examination results of antibody levels were sorted from those with the lowest to highest value. The antibody levels were divided into three groups (low (<3), moderate (>300), and high (>600)). The low antibody levels group was randomised and one sample was taken to be tested for neutralisation, representing the group. Likewise, for the moderate and high groups, one sample was taken each to be tested for neutralisation. The three groups of antibody levels were divided into two major groups: the antibody group of subjects who consumed probiotics and did not consume probiotics.

## 3 Experimental design

There were 12 neutralisation test groups in this study: A1 group (no probiotics, no spike protein SARS-CoV-2, IgG antibody <3), A2 group (no probiotics, no spike protein SARS-CoV-2, IgG antibody 325.76), A3 group (no probiotics, no spike protein SARS-CoV-2, IgG antibody 646.18), B1 group (no probiotics, with spike protein SARS-CoV-2, IgG antibody <3), B2 group (no probiotics, with spike protein SARS-CoV-2, IgG antibody 325.76), B3 group (no probiotics, with spike protein SARS-CoV-2, IgG antibody 646.18), C1 group (with probiotics, no spike protein SARS-CoV-2, IgG antibody <3), C2 group (with probiotics, no spike protein SARS-CoV-2, IgG antibody 325.76), C3 group (with probiotics, no spike protein SARS-CoV-2, IgG antibody 646.18), D1 group (with probiotics, with spike protein SARS-CoV-2, IgG antibody <3), D2 group (with probiotics, with spike protein SARS-CoV-2, IgG antibody 325.76), and D3 group (with probiotics, with spike protein SARS-CoV-2, IgG antibody 646.18). There were seven samples in each group.

### 3.1 Neutralisation test

PBMCs from healthy volunteers (negative result after polymerase chain reaction (PCR) examination) were isolated for 1 h and grown in a Roswell Park Memorial Institute (RPMI) 1640 medium, supplemented by fetal bovine serum. The cell was cultured in incubators set at 37 °C with 5% CO_2_ and passaged every 2–3 days. A neutralisation test was carried out on PBMC cultures modelled for COVID-19 by giving the intervention of recombinant SARS-CoV-2 spike protein S1 produced by Raybiotech USA, cultured for three days. A neutralisation test was carried out by administering 100 μL of antibodies to each appropriate group. After 72 h, the supernatant of the cells was harvested for LDH and ATP measurement. In addition, cell viability was also examined using the XTT method.

### 3.2 LDH measurement

According to the manufacturer's instructions, cell cytotoxicity was determined with the LDH cytotoxicity assay kit by Elabscience.

#### 3.2.1 Standard curve creation

A standard pyruethic acid solution of 2 mol/mL was diluted with distilled water with serial concentrations of 0, 0.05, 0.1, 0.2, 0.4, 0.6, 0.8 and 1 mol/mL.

#### 3.2.2 Sample measurement

In standard wells: 5 L of distillate water and a standard solution of pyruvic acid with different concentrations were added.

In the sample well, 20 μL of the sample was added while 5 μL of ddH_2_O water and 20 μL of the sample were added to the control wells. Then, 5 μL of reagent one was added to each well, while 5 μL of reagent two was added to the sample wells.

All solutions were mixed thoroughly and incubated at 37 °C for 15 min. All wells were combined with 25 μL of reagent three and incubated at 37 °C for 15 min. After incubation, 200 μL of reagent four was poured into each well. The solution was mixed thoroughly and left at room temperature for 5 min. All wells were read at a wavelength of 450 nm.

The LDH was calculated according to the following equations:

$$\text{LDH activity }\left(\frac{\text{U}}{\text{gprot}}\right)=\left(\text{A}450-\text{b}\right)+a\;\times f+Cprot\times 1000$$

### 3.3 ATP measurement

The Elabscience ATP Assay Kit was used for the ATP measurements. ATP measurements were conducted at the Physiology Laboratory of the Faculty of Medicine at Universitas Brawijaya. Three tubes were prepared before measurement. Tube blanks: 30 μL of the sample was transferred into a 1.5 mL sterile tube, then combined with 330 μL of control working solution. Control tube: 1 mmol/L standard ATP was taken at 30 μL, plus 330 μL of detections working solution. Sample tube: 30 μL of the sample was taken into a 1.5 mL sterile tube, then mixed with 330 μL of control working solution.

The suspension was mixed and incubated at 37 °C for 30 min, and 50 L of reagent was added to each tube. The rest was mixed with a vortex for 3 s, then rotated 1000 g for 5 min, after which 300 L of supernatant was taken. The supernatant was added to 500 L of chromogen in each tube. The mixed suspension was left for 2 min at room temperature. 500 μL of reagent 8 was added to each tube. The suspension was mixed and allowed to stand for 5 min at room temperature. After incubation, it was read with a spectrophotometer at 635 nm.

Measurement formula

$$\text{ATP}=\frac{ODsample-ODcontrol}{ODstandard-ODblank}\;\;\times \text{c / n / V}2\times \text{f}$$

### 3.4 Cell viability assay

An XTT cell viability kit manufactured by Biotium was used to determine the number of live cells after exposure to SARS-CoV-2 spike recombinant protein (S1 subunit; produced by Raybiotech, USA). Firstly, the cells were transferred into 96-well tissue culture plates. Cells were seeded at densities between 5000 and 10,000 cells per well to reach optimal density within 48–72 h. The final volume of culture medium in each well was 100 μL, and the medium contained up to 10% serum. The working solution was prepared beforehand. Then, 25 μL of activation reagent and 5 mL of XTT solution were mixed into each 96-well plate to be tested to derive activated XTT solution. After that, 25 μL of the activated XTT solution was added to the 100 μL medium in each well. Samples were then incubated in an incubator for 2–5 h The plate was shaken gently to distribute the dye in the wells. The samples’ absorbance signal was measured with a spectrophotometer at a wavelength of 450–500 nm. Changes in cellular morphology were observed using an inverted microscope (Olympus CKX41), and images were taken using a digital camera.

### 3.5 Statistical analysis

All of the data values were analyzed with SPSS software version 21. A one-way ANOVA test was used to determine the differences between groups. The statistical significance was set at p < 0.05.

## 4 Results

### 4.1 IgG sRBD levels

The sRBD examination was carried out on two groups of COVID-19 sufferers which were divided into 2 groups: the group consuming probiotics and group not consuming probiotics on day 21 after being tested negatif for COVID-19. The independent T-test was used to compare the mean difference between the control and intervention groups. The analysis results showed no significant difference (p = 0.421) in IgG sRBD levels in the control and intervention groups **(**Supplementary material 1).

### 4.2 Lactate dehydrogenase (LDH) activity

The different test results were derived using the analysis of variance (ANOVA) for data that were normally distributed. A non-parametric difference test was also carried out using the Kruskal Wallis test for all groups to test their difference in the values against the A1 group, whose data were not normally distributed. The analysis results (Supplementary material 2) showed that the two types of tests obtained p-values of <0.001, which indicate significant difference between the treatment groups.

**Fig. 1:**
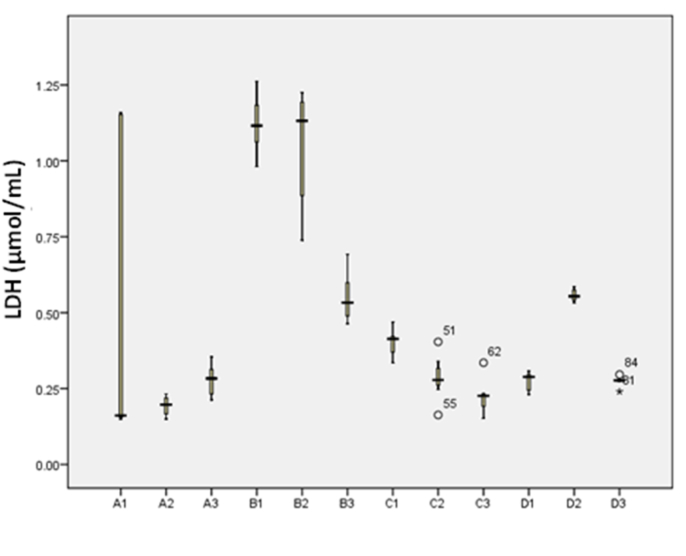
Boxplot of Lactate Dehydrogenase (LDH) activity in 12 groups.

Based on the boxplot results (Fig. 1), the data distribution shows that group A1 had an extensive range of data that intersected with data in other groups. LDH appeared to be the highest in groups B1, B2, and B3, without probiotics and infection (+). In addition to these three groups, there was one more group that had a relatively high LDH value, namely group D2 (probiotic (+), infection (+), with antibody level of 325.76.

### 4.3 ATP activity

The analysis results showed that the ATP data obtained were normally distributed apart from group A3. The highest mean and median ATP values were obtained in group D2, and the lowest mean and median values were obtained in group B2. The results of the different tests were carried out using ANOVA for data that were normally distributed. A non-parametric difference test was also carried out using the Kruskal Wallis test for the entire group to test the difference in their values against the A3 group whose data were not normally distributed. The analysis results (Supplementary material 3) showed that the two types of tests obtained p-values < 0.001, or 0.05, which mean that there were significant differences between the treatment groups.

**Fig. 2:**
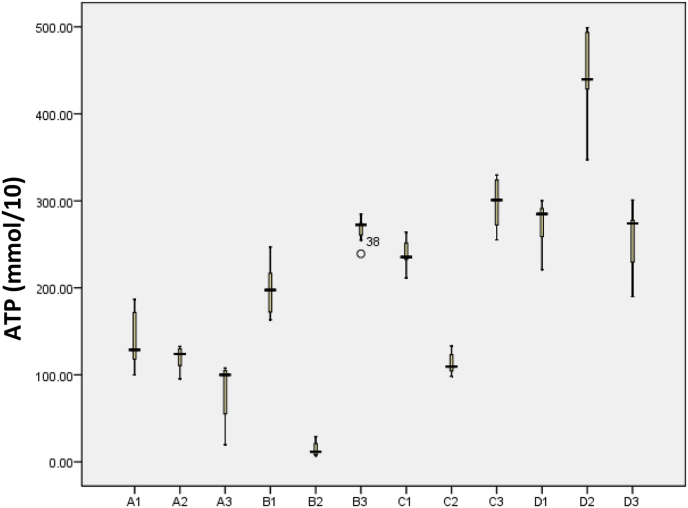
Boxplot of ATP activity in 12 groups.

The data distribution based on boxplot results (Fig. 2) shows that group D2 had the highest value and tended not to intersect with data in other groups. In addition to the D2 group, the ATP values of the C3, D1, and D3 groups were the highest. The lowest ATP activity values were seen in group B2, followed by group A3.

### 4.4 Effect of LDH activity on ATP

The analysis of LDH activity on ATP (Table 1) showed no significant effect (p = 0.238). Although the effect was not significant, the direction of the effect appeared to be negative, as indicated by the unstandardized coefficient value (β = −44.543), suggesting a tendency of decreased ATP when there was an increase in LDH. Overall, it can be concluded that the interim results of the analysis showed LDH activity has not provided evidence of a significant effect on the current decrease in ATP activity.

**Table 1:** Analysis of the effect of LDH activity on ATP (n = 84).

No	Independent Variable	Dependent Variable	β	p^a^	R^2^	Comment
1	LDH	ATP	−44.543	0.238	0.017	No significant difference

a Linear regression test.

### 4.5 Cell viability

**Fig. 3:**
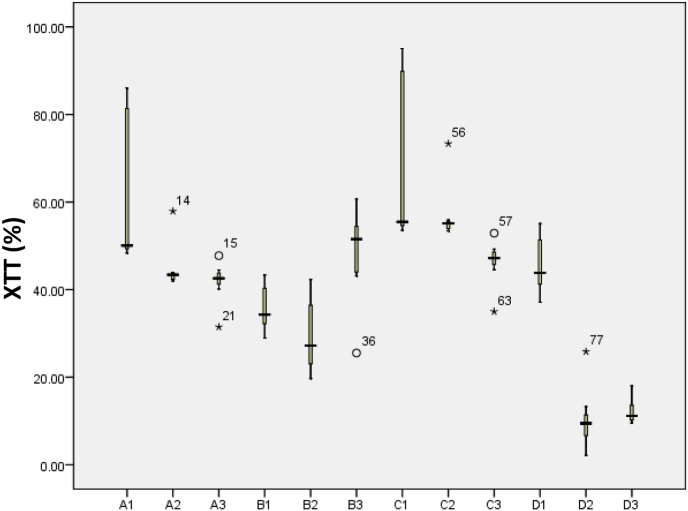
Boxplot of XTT assay results in each group.

The post hoc test results (Supplementary material 7) showed that groups D2 and D3 were the groups that had the most differences in scores compared to other groups, and between groups D2 and D3, the XTT values were not significantly different (Fig. 3).

The LDH and ATP activity analysis had a significant effect on XTT (Table 2). The effect had a negative direction, indicated by the unstandardized coefficients value, having a tendency of decreased XTT (cell viability %) when there was an increase in LDH and ATP. The analysis results can conclude that the activity of LDH and ATP provide evidence of a significant effect on the decrease in XTT activity (cell viability %).

**Table 2:** Analysis of the effect of LDH and ATP activities on XTT.

No	Independent Variable	Dependent Variable	β	p^a^	R^2^
1	LDH	XTT	−16.827	0.006	0.14
2	ATP		−0.055	0.003	

a Linear regression test.

## 5 Discussion

PBMCs chosen are a popular tissue for immunological, molecular, and pharmacogenomic research. PBMCs are diverse cells with a round nucleus (i.e., lymphocytes, monocytes, natural killer cells (NK cells), or dendritic cells) derived from peripheral blood. PBMCs have been demonstrated to be susceptible to SARS-CoV infection and promote viral replication [[7], [8], [9], [10]]. Human peripheral blood mononuclear cells (PBMCs) are peripheral blood cells carrying a single round nucleus. PBMCs comprise several classes of immune cells, including T cells, B cells, monocytes, dendritic cells, and natural killer (NK) cells [11,12].

This diverse group of immune cells collaborates to keep humans safe from infections. Because they operate as a line of defense against infection and disease, their placement in peripheral blood is important. Isolation of PBMCs from whole blood samples is the most common method [13].

The identification and development of novel COVID-19 therapeutics will be aided by a better understanding of the SARS-CoV-2 virus's infection pathways, virus-host protein interactions, and mechanisms of virus-induced cytopathic effects [14]. To simulate this, SARS-CoV-2 virus-like protein was given into PBMC cultures and assessed after 72 h. An antibody neutralisation test was performed on the COVID-19 survivors who were fed probiotics and non-probiotics.

The rationalization for the neutralisation test using the probiotic survivor group's antibodies is that there may be differences in the ability of these antibodies to prevent cytotoxic and cytopathic cells from occurring. Theoretically, there is a balanced regulation of cell death with new cell growth. If cell death incidence is very high, there will be an imbalance in the body that results in organ damage. This study's results show that it is not only the role of probiotics that causes an increase or decrease in cytotoxic, cytopathic, and cell viability. Antibody levels also have an important role in the results of this study.

In this study, there was a good proliferation of PBMC cultures, but unfortunately, a detailed examination of which cells were affected was not performed. The general extent of damage to cells was observed by examining the levels of LDH and ATP from the cell culture supernatants subjected to neutralisation assays.

Cytotoxicity degree was measured by LDH release. LDH is released from the cell's cytoplasm to the culture medium due to cell membrane damage and cell lysis. The measurement was based on an assessment of LDH's ability to oxidise lactic acid to pyruvic acid, which is dependent on the increase of the release level. The rise of LDH activity in the cell cultures' supernatants showed a relationship with the dead cell percentage (increased cytotoxicity correlated with the increasing dead cell content) [15]. A case report written by Yi Han et al. (2020) states that LDH is one of the independent prediction risks for the severity of COVID-19 [16]. There may be a relationship between previous works and this study's results, but it cannot be concluded that probiotics are good for preventing the increase in LDH.

The mechanism underlying how the antibody in this study can manipulate cells by checking IgG sRBD levels has not been explained in detail. Normal human IgG has anti-inflammatory and immunomodulatory properties well demonstrated *in vivo* and *in vitro* [17]. A study conducted by Ronda et al. (2003) states that IgG could inhibit TNF alpha secretion, which is activated by macrophages. The results of our study may also be due to the effect of antibodies that can reduce pro-inflammatory so that it indirectly inhibits apoptosis, as shown in the LDH box plot. The boxplot (Fig. 1) shows that groups B1 and B2 have the highest levels significantly compared to the other groups. It is possible that this process occurs through a mechanism of inhibition of proinflammatory reduction due to the administration of probiotics and antibody levels in this group.

Interestingly, in the analysis of ATP levels, group D2 had the highest ATP levels while group B2 had the lowest ATP levels. It is hypothesised that the engulfing process by macrophages against infected cells is likely to be more active in the COVID-19 model group tested for neutralisation with IgG survivors taking probiotics. ATP is released by dying cells. In a study conducted by Martins et al. (2014), it was found that immunogenic cell death activation was activated by the release of ATP through ER stress, caspase activation, and autophagy in cancer cells given anti-cancer drugs [18]. Further research needs to be done to determine whether there is a relationship between probiotic consumption and increased ATP release. Based on the data analysis results in this study, it seems that there was a relationship between ATP and LDH results. There was a tendency of decreased ATP when there was an increase in LDH, although the statistical analysis shows a small R square (Table 1). Regarding the results of this statistical analysis, there are many possibilities that could be the cause, because the sample size was small or the data range was too wide between groups.

The hypothesis above is also supported by studies conducted by Adeniji et al. (2021); there are possible theories of antibody-dependent complement deposition (ADCD), antibody-dependent cell-mediated phagocytosis (ADCP), and antibody-dependent cell-mediated cytotoxicity (ADCC) that occurred in this study's *in vitro* experiments. It also stated that anti-S1 and anti-RBD antibodies could cause ADCP, ADCD, and ADCC. These functions are beneficial as they contribute to pathogen clearance; however, they also can induce inflammation. It cannot be explained clearly whether the phenomenon of ADCP, ADCD, or ADCC occurred in this study [14].

Higher titers of SARS-CoV-2-specific neutralising antibodies have been associated with higher COVID-19 severity [19]. Antibodies can elicit a variety of Fc-mediated innate immunological responses, including antibody-dependent complement deposition (ADCD), antibody-dependent cellular phagocytosis (ADCP), and antibody-dependent cell-mediated cytotoxicity (ADCC). These innate immune processes are important because they help to eliminate pathogens, but they can also cause inflammation during viral infections [14].

High levels of neutralising antibodies are assumed to be associated with the innate immune response or strong inflammation overreaction. They do not necessarily play a protective role in the SARS-CoV-2 infection [14]. This may explain the phenomenon that our results showed that the D3 group showed low cell viability even though it was a group with high antibody levels (Fig. 3).

The changes in cell morphology caused by infecting viruses are called cytopathic effects (CPE). LDH is released from the cell's cytoplasm to the culture medium due to cell membrane damage and lysis. The measurement is based on an assessment of the LDH's ability to oxidise lactic acid to pyruvic acid, which is dependent on the increase of the release level. The rise of LDH activity in the cell cultures' supernatants shows a relationship with the dead cell percentage. Increased cytotoxicity correlates with the increasing content of dead cells [15]. If we look at the LDH boxplot (Fig. 1), it is unique that there was a very high increase in LDH levels in groups B1 and B2, followed by group B3, where all three were considered infected and did not consume probiotics. From this phenomenon, we can simply assume that the administration of probiotics will have a good effect in inhibiting the increase in LDH. However, in group D2 where this group is the group that is considered infected and consuming probiotics, they also have higher LDH levels than groups D1 and D2. This raises an interesting question whether there is a relationship between excessively high antibody levels and consumption of probiotics actually causing a less than optimal effect on cell conditions (when compared to groups D1 and D2). The condition in the D2 group is the condition that we say is the worst supported by the ATP boxplot data (Fig. 2) where the D2 group is also the group with the highest ATP levels.

The LDH and ATP activity analysis results have a significant effect on XTT (cell viability %). The results of the cell viability test that we show in the boxplot image (Fig. 3), group D2 has the worst cell viability results. However, there was no significant difference in cell viability between groups D2 and D3. Based on the explanation that we have written above, it is true that this D2 group has the highest levels of LDH and ATP. Meanwhile, the group with the highest cell viability occurred in the C1 group (consumption of probiotics, without infection with antibody levels <3). This phenomenon can be an inspiration for clinical research as a further proof that probiotics consumed as a preventive measure and maintain cell viability may provide good outcomes in the world of health.

**Fig. 4:**
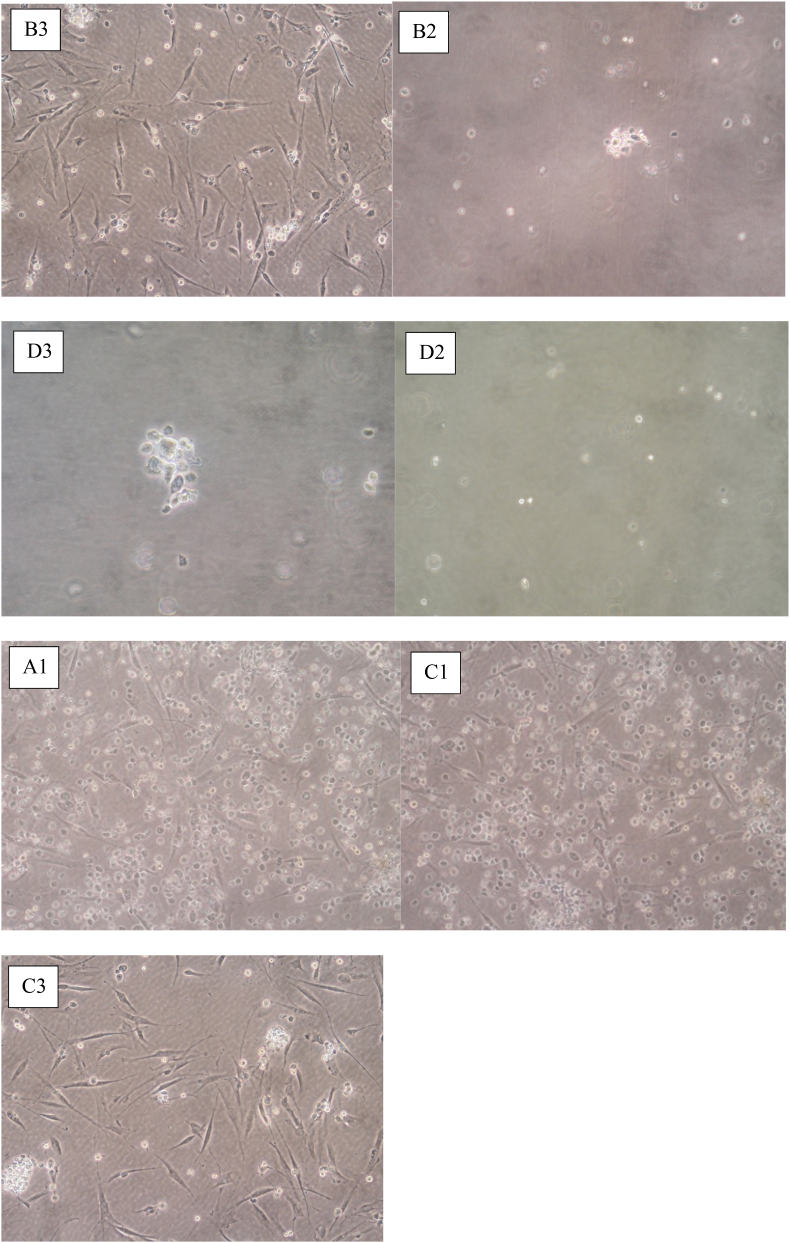
Cell viability of the B3, B2, D3, D2, A1, C1, C3 group observed under inverted microscope (Olympus CKX41) with 40× magnification.

The conclusion drawn from the examination of cell viability in this study is that LDH and ATP activities provide evidence of a significant effect on decreasing XTT activity (cell viability %). These results are supported by previous studies discovering relationships between high LDH and ATP levels on cell viability [20]. Representative microscopic images of cell viability (Fig. 4) showed the morphology of dead cells in control group and intervention group.

However, the following limitations to this study must be noted. First, the ELISA assay was not used to evaluate specific antibodies in the serum. Second, the cell death evaluation during infection was only tested based on LDH and ATP levels. Thus, it should be quantified using flow cytometry to better evaluate its predicting amounts of dead cells. Third, since probiotic consumption of COVID patients was not monitored regularly, the effect of probiotics on antibody titer may not have been explained well. Fourth, the type and amount of daily food consumption by the two groups of subjects (probiotic and non-probiotic) were not examined in this study. Fifth, there are several phenomena occurred in this study that we cannot explain with good scientific reasons.

## 6 Conclusion

We have not been able to draw firm conclusions from the results of this study. However, based on our results, there is a possibility that probiotics may have a good effect on cell viability when consumed in uninfected conditions and low antibody levels. Some things that can be considered for further research are optimal levels of sRBD to fight viral infections and the provision of probiotics in infectious conditions.

## Ethics approval and consent to participate

This research has been granted ethical approval from the Ethics Committee of Dr. Soetomo General Academic Hospital, with the ethical conduct number: 0117/LOE/301.4.2/IX/2020.

## Author contributions statement

Anna Surgean Veterini: Conceptualization, Methodology, Writing - Original Draft, Writing - Review & Editing, Formal Analysis. Cita Rosita Sigit Prakoeswa: Project administration, Supervision, Validation. Damayanti Tinduh: Supervision, Validation. Satuman: Investigation, Resources.

## Funding

This study was supported by research grant from PT. IDS Medical Systems Indonesia.

## Declaration of competing interest

The authors declare that they have no known competing financial interests or personal relationships that could have appeared to influence the work reported in this paper.

## Footnotes

Appendix A Supplementary data to this article can be found online at https://doi.org/10.1016/j.bbrep.2022.101348.
