# Supplementary material for: Simple neutralization test report: Do probiotics contribute to COVID-19 therapy?
Source: Biochem Biophys Rep. 2022 Sep 13;32:101348. doi: 10.1016/j.bbrep.2022.101348 (PMC9468316; doi:10.1016/j.bbrep.2022.101348)
Supplement: Multimedia component 1 [file mmc1.pdf]

PEMERINTAH PROPINSI JAWA TIMUR
RUMAH SAKIT UMUM DAERAH Dr. SOETOMO
KOMITE ETIK PENELITIAN KESEHATAN

Jl. Mayjen Prof. Dr. Moestopo No. 6-8, Telp. 5501164
SURABAYA 60286

Kepada Yth : Ka. SMF Anestesiologi
Dari : Ka. Komite Etik Penelitian Kesehatan
Nomor : 159 /113/Komitlitkes/ XII /2020
Tanggal : 07 DEC 2020
Lampiran : -
Perihal : Persetujuan Amandemen Penelitian

Menindaklanjuti surat permohonan saudara No: 354 / IX / 2020 tanggal 09 November 2020, mengenai permohonan amandemen penelitian. Dengan ini diberitahukan bahwa Komite Etik Penelitian Kesehatan RSUD Dr. Soetomo Surabaya telah mempelajari dengan seksama permohonan amandemen penelitian tersebut dengan peneliti utama Dr. Anna Surgean Veterini dr., Sp.An.KIC *dan judul penelitian yaitu:*

"Pengaruh Pemberian Probiotik Terhadap Perubahan Daya Hidup dan Daya Rusak Virus SARS COV-2 pada Kultur Sel"

Terdapat perubahan judul penelitian yaitu:

Adendum	Awal	Menjadi
Perubahan judul penelitian	Pengaruh Pemberian Probiotik Terhadap Perubahan Daya Hidup dan Daya Rusak Virus SARS COV-2 pada Kultur Sel	Pengaruh Konsumsi Probiotik Terhadap Kadar Antibodi Survivor Covid-19 (Uji Netralisasi Antibodi Pada Sel Kultur yang Diinfeksi dengan Isolat Virus Sars Cov-2)
Perubahan rancangan penelitian	Eksperimental pada Sel Kultur	Eksperimental pemberian probiotik pada Tenaga Medis di RSUD Dr. Soetomo Kemudian dilakukan pemeriksaan titer antibodi serum. Selanjutnya dilakukan uji netralisasi pada sel kultur dan atau PBMC
Perubahan lokasi penelitian	Tropical Disease Center Universitas Airlangga	RSUD Dr. Soetomo dan Tropical Disease Center Universitas Airlangga
Perubahan waktu penelitian	3 Bulan	Januari 2021-Desember 2021 (1Tahun)

Pada dasarnya kami tidak keberatan dan menyetujui adanya amandemen penelitian tersebut. Selanjutnya kami mengharapkan kepada peneliti untuk selalu melaporkan perkembangan penelitian tersebut kepada Komite Etik Penelitian Kesehatan RSUD Dr. Soetomo Surabaya.

Atas perhatiannya kami sampaikan terimakasih.

Ketua Komite Etik Penelitian Kesehatan
RSUD Dr. Soetomo Surabaya

RSUD Dr. Soetomo Surabaya — Komite Etik Penelitian Kesehatan

Dr. Elizeus Hanindito, dr., Sp.An., KIC., KAP
NIP. 19511007 197903 1 002

Tembusan kepada Yth,

1. Direktur Utama RSUD Dr. Soetomo Surabaya
2. Drektur Pendidikan Profesi dan Penelitian
3. Kepala Bidang Litbang
4. Ketua *Clinical Research Unit (CRU)*
5. Yang bersangkutan

Scanned by TapScanner

PEMERINTAH PROPINSI JAWA TIMUR
RUMAH SAKIT UMUM DAERAH Dr. SOETOMO
KOMITE ETIK PENELITIAN KESEHATAN
Jl. Mayjen Prof. Dr. Moestopo No. 6-8, Telp. 031-5501071-5501073, Fax. 031-5501164
SURABAYA 60286

SURAT EXEMPTION

(" LETTER OF EXEMPTION ")

Ref. No. : 0117/LOE/301.4.2/IX/2020

Judul Protokol Penelitian	:	PENGARUH KONSUMSI PROBIOTIK TERHADAP KADAR ANTIBODI SURVIVOR COVID-19 (Uji Netralisasi Antibodi pada Sel Kultur yang Diinfeksi dengan Isolat Virus SARS COV-2)
Dokumen yang disetujui	:	0198/119/IX/2020 (versi: 2)
Peneliti Utama	:	Dr. Anna Surgean Veterini, dr., Sp.An.KIC
Peneliti Lain	:	1. Prof. Subijanto Marto Soedarmo, dr., Sp.A (K)<br>2. Prof. Dr. Cita Rosita Sigit Prakoeswa, dr., SpKK (K)., FINSDV., FAADV<br>3. Dr. Hamzah,, dr., Sp.An., KNA., KIC<br>4. Prof. Dr. Nancy Margarita Rehatta, dr., Sp.An., KIC., KNA<br>5. Dr. Damayanti Tinduh, dr., Sp.KFR (K)<br>6. Dr. Eighty Mardiyan Kurniawati, dr., Sp.OG (K)
Instalasi/Tempat Penelitian	:	SMF Anestesiologi<br>RSUD Dr. Soetomo

Komite Etik Penelitian Kesehatan RSUD Dr Soetomo menyatakan bahwa dokumen diatas sesuai dengan The Office for Human Research Protections (OHRP) dibawah persyaratan the U.S. Department of Health and Human Services (HHS) Regulasi 45 CFR bagian 46 untuk **exempt review**.

RSUD Dr. Soetomo Komite Etik Penelitian Kesehatan SURABAYA

Dr. Dominicus Husada dr., SpA(K)
Ketua Panel 4

Dr. Evelyn Komaratih dr., SpM(K)
Sekretaris Panel 4

Scanned by TapScanner
