# Supplementary material for: Simple neutralization test report: Do probiotics contribute to COVID-19 therapy?
Source: Biochem Biophys Rep. 2022 Sep 13;32:101348. doi: 10.1016/j.bbrep.2022.101348 (PMC9468316; doi:10.1016/j.bbrep.2022.101348)
Supplement: Multimedia component 2 [file mmc2.docx]

**Supplementary**

**Supplementary material 1**. IgG sRBD level and Independent T test of control and probiotics group

| **No** | **Group** | **Test** | **IgG**  **Level** | **Unit** | **Subject** | **Antibody test day-** |
| --- | --- | --- | --- | --- | --- | --- |
| 1 | Control | nCoV S-RBD | <3 | U/mL | RIAN NOFIANSYAH, dr | 10 |
| 2 |  | nCoV S-RBD | 285.75 | U/mL | ARDI GUSTIAN, dr | 13 |
| 3 |  | nCoV S-RBD | 208.2 | U/mL | Dr. DHIKRULLOH ANWAR | 20 |
| 4 |  | nCoV S-RBD | 441.18 | U/mL | Juanita Tetengean | 21 |
| 5 |  | nCoV S-RBD | 342.22 | U/mL | Nanda Bagus Pratikno | 20 |
| 6 |  | nCoV S-RBD | 120.19 | U/mL | Huwaida Nabilah | 21 |
| 7 |  | nCoV S-RBD | <3 | U/mL | Citra AULIA BACHTIAR | 21 |
| 8 |  | nCoV S-RBD | <3 | U/mL | Indra Mahyul | 21 |
| 9 |  | nCoV S-RBD | 630.85 | U/mL | Siti Asror | 21 |
| 10 |  | nCoV S-RBD | 222.72 | U/mL | Yusriandi Rachman | 21 |
| 11 |  | nCoV S-RBD | 125.98 | U/mL | Ade Anugrah | 21 |
| 12 |  | nCoV S-RBD | 343.25 | U/mL | Ida Ayu | 20 |
| 13 |  | nCoV S-RBD | 646.18 | U/mL | Qurrotul R | 21 |
| 14 |  | nCoV S-RBD | 214.88 | U/mL | Ririn Ria Ayu L | 22 |
| 15 |  | nCoV S-RBD | 32.57 | U/mL | Widodo | 23 |
| 16 | Probiotics | nCoV S-RBD | <3 | U/mL | Dr. HIDAJAT PRASOJO FAQIH | 17 |
| 17 |  | nCoV S-RBD | 325.76 | U/mL | WIKAN PURWITOHANTORO S, Ns.Kep | 25 |
| 18 |  | nCoV S-RBD | 134.49 | U/mL | Ridiyani | 15 |
| 19 |  | nCoV S-RBD | 108.32 | U/mL | Dr. Manggala Pasca Wardhana, Sp.OG (K) | 17 |
| 20 |  | nCoV S-RBD | 520.24 | U/mL | ADDE MIDIAN PUTRA CHAFID | 18 |
| 21 |  | nCoV S-RBD | 69.45 | U/mL | Dr. KHOIRUNNISA NOVITASARI | 21 |
| 22 |  | nCoV S-RBD | <3 | U/mL | Hanung Aryana | 20 |
| 23 |  | nCoV S-RBD | <3 | U/mL | Victor Lamerkabel | 16 |
| 24 |  | nCoV S-RBD | <3 | U/mL | Antonius Abimasetyo Putro | 16 |
| 25 |  | nCoV S-RBD | 285.16 | U/mL | Idha Kanyawardhani | 20 |
| 26 |  | nCoV S-RBD | 285.6 | U/mL | Lucky Andriyanto | 20 |
| 27 |  | nCoV S-RBD | 146.45 | U/mL | Mahisa Brahmana | 15 |
| 28 |  | nCoV S-RBD | 88.55 | U/mL | Riska Wahyuningtyas | 21 |
| 29 |  | nCoV S-RBD | 633.35 | U/mL | Rahma Wulan Pratiwi S | 20 |
| 30 |  | nCoV S-RBD | 108.96 | U/mL | Dini Septian | 14 |

| **Group** | **IgG Level** | |
| --- | --- | --- |
|  | **Mean** | **p (T test)** |
| Control | 241.5187 | 0.421 |
| Probiotics | 181.2220 |  |

**Supplementary material 2.** LDH Activity in 12 groups

| **Group** | **LDH Activity** | | | | | | | |
| --- | --- | --- | --- | --- | --- | --- | --- | --- |
|  | **Mean** | **Median** | **SD** | **Min.** | **Maks.** | **p*** | **p**** | **p***** |
| A1 | 0.58 | 0.16 | 0.53 | 0.15 | 1.16 | 0.002 | <0.001 | <0.001 |
| A2 | 0.19 | 0.20 | 0.03 | 0.15 | 0.23 | 0.442 |  |  |
| A3 | 0.28 | 0.28 | 0.05 | 0.21 | 0.35 | 0.556 |  |  |
| B1 | 1.12 | 1.12 | 0.10 | 0.98 | 1.26 | 0.958 |  |  |
| B2 | 1.04 | 1.13 | 0.20 | 0.74 | 1.22 | 0.180 |  |  |
| B3 | 0.55 | 0.53 | 0.09 | 0.46 | 0.69 | 0.286 |  |  |
| C1 | 0.40 | 0.41 | 0.05 | 0.34 | 0.47 | 0.603 |  |  |
| C2 | 0.29 | 0.28 | 0.07 | 0.16 | 0.40 | 0.893 |  |  |
| C3 | 0.22 | 0.23 | 0.06 | 0.15 | 0.34 | 0.281 |  |  |
| D1 | 0.27 | 0.29 | 0.03 | 0.23 | 0.31 | 0.162 |  |  |
| D2 | 0.56 | 0.55 | 0.02 | 0.53 | 0.58 | 0.310 |  |  |
| D3 | 0.27 | 0.28 | 0.02 | 0.24 | 0.30 | 0.095 |  |  |

p* = Shapiro-Wilk test; p** = Anova test; p*** = Kruskal Wallis test

**Supplementary material 3.** Descriptive Data, Normality Test, and Different Test of ATP Activity in 12 groups

| **Group** | **ATP** | | | | | | | |
| --- | --- | --- | --- | --- | --- | --- | --- | --- |
|  | **Mean** | **Median** | **SD** | **Min.** | **Maks.** | **p*** | **p**** | **p***** |
| A1 | 142.15 | 34.39 | 128.66 | 99.79 | 186.71 | 0.382 | <0.001 | <0.001 |
| A2 | 118.98 | 14.50 | 124.00 | 95.37 | 132.63 | 0.184 |  |  |
| A3 | 78.21 | 39.44 | 100.00 | 19.79 | 107.79 | 0.007 |  |  |
| B1 | 197.96 | 30.72 | 197.47 | 163.37 | 246.74 | 0.599 |  |  |
| B2 | 15.21 | 9.12 | 11.65 | 6.95 | 28.95 | 0.100 |  |  |
| B3 | 266.53 | 15.09 | 272.42 | 239.16 | 284.63 | 0.449 |  |  |
| C1 | 239.88 | 17.93 | 235.37 | 211.37 | 264.00 | 0.636 |  |  |
| C2 | 113.75 | 12.78 | 109.41 | 98.29 | 133.17 | 0.562 |  |  |
| C3 | 296.90 | 30.69 | 301.05 | 255.16 | 329.68 | 0.287 |  |  |
| D1 | 272.36 | 28.02 | 285.05 | 221.05 | 300.21 | 0.315 |  |  |
| D2 | 447.22 | 54.49 | 439.58 | 347.37 | 498.95 | 0.211 |  |  |
| D3 | 254.20 | 39.40 | 274.11 | 189.90 | 301.05 | 0.534 |  |  |

p* = Shapiro-Wilk test; p** = Anova test ; p*** = Kruskal Wallis test

**Supplementary material 4.** Post Hoc Test of Lactate Dehydrogenase (LDH) Activity in 12 Treatment Groups

| **Group** | **A1** | **A2** | **A3** | **B1** | **B2** | **B3** | **C1** | **C2** | **C3** | **D1** | **D2** | **D3** |
| --- | --- | --- | --- | --- | --- | --- | --- | --- | --- | --- | --- | --- |
| A1 |  | 1.000* | 0.710* | 0.128* | 0.128* | 0.710* | 0.710* | 0.710* | 0.902* | 0.710* | 0.710* | 0.710* |
| A2 | 1.000* |  | 0.107 | 0.000 | 0.000 | 0.000 | 0.000 | 0.230 | 0.969 | 0.014 | 0.000 | 0.005 |
| A3 | 0.710* | 0.107 |  | 0.000 | 0.001 | 0.001 | 0.019 | 1.000 | 0.761 | 1.000 | 0.000 | 1.000 |
| B1 | 0.128* | 0.000 | 0.000 |  | 0.988 | 0.000 | 0.000 | 0.000 | 0.000 | 0.000 | 0.000 | 0.000 |
| B2 | 0.128* | 0.000 | 0.001 | 0.988 |  | 0.007 | 0.002 | 0.000 | 0.000 | 0.001 | 0.011 | 0.001 |
| B3 | 0.710* | 0.000 | 0.001 | 0.000 | 0.007 |  | 0.055 | 0.002 | 0.000 | 0.001 | 1.000 | 0.002 |
| C1 | 0.710* | 0.000 | 0.019 | 0.000 | 0.002 | 0.055 |  | 0.118 | 0.002 | 0.003 | 0.001 | 0.004 |
| C2 | 0.710* | 0.230 | 1.000 | 0.000 | 0.000 | 0.002 | 0.118 |  | 0.785 | 1.000 | 0.001 | 1.000 |
| C3 | 0.902* | 0.969 | 0.761 | 0.000 | 0.000 | 0.000 | 0.002 | 0.785 |  | 0.663 | 0.000 | 0.535 |
| D1 | 0.710* | 0.014 | 1.000 | 0.000 | 0.001 | 0.001 | 0.003 | 1.000 | 0.663 |  | 0.000 | 1.000 |
| D2 | 0.710* | 0.000 | 0.000 | 0.000 | 0.011 | 1.000 | 0.001 | 0.001 | 0.000 | 0.000 |  | 0.000 |
| D3 | 0.710* | 0.005 | 1.000 | 0.000 | 0.001 | 0.002 | 0.004 | 1.000 | 0.535 | 1.000 | 0.000 |  |

Posthoc test using Games-Howell test, except (*) using Mann-Whitney test

**Supplementary material 5.** Post Hoc Test of ATP Activity in 12 Treatment Groups

| **Group** | **A1** | **A2** | **A3** | **B1** | **B2** | **B3** | **C1** | **C2** | **C3** | **D1** | **D2** | **D3** |
| --- | --- | --- | --- | --- | --- | --- | --- | --- | --- | --- | --- | --- |
| A1 |  | 0.829 | 0.007* | 0.148 | 0.001 | 0.000 | 0.002 | 0.632 | 0.000 | 0.000 | 0.000 | 0.003 |
| A2 | 0.829 |  | 0.026* | 0.005 | 0.000 | 0.000 | 0.000 | 0.999 | 0.000 | 0.000 | 0.000 | 0.001 |
| A3 | 0.007* | 0.026* |  | 0.001* | 0.007* | 0.001* | 0.001* | 0.038* | 0.001* | 0.001* | 0.001* | 0.001* |
| B1 | 0.148 | 0.005 | 0.001* |  | 0.000 | 0.012 | 0.186 | 0.003 | 0.002 | 0.013 | 0.000 | 0.209 |
| B2 | 0.001 | 0.000 | 0.007* | 0.000 |  | 0.000 | 0.000 | 0.000 | 0.000 | 0.000 | 0.000 | 0.000 |
| B3 | 0.000 | 0.000 | 0.001* | 0.012 | 0.000 |  | 0.198 | 0.000 | 0.476 | 1.000 | 0.001 | 0.998 |
| C1 | 0.002 | 0.000 | 0.001* | 0.186 | 0.000 | 0.198 |  | 0.000 | 0.040 | 0.360 | 0.000 | 0.996 |
| C2 | 0.632 | 0.999 | 0.038* | 0.003 | 0.000 | 0.000 | 0.000 |  | 0.000 | 0.000 | 0.000 | 0.001 |
| C3 | 0.000 | 0.000 | 0.001* | 0.002 | 0.000 | 0.476 | 0.040 | 0.000 |  | 0.871 | 0.003 | 0.509 |
| D1 | 0.000 | 0.000 | 0.001* | 0.013 | 0.000 | 1.000 | 0.360 | 0.000 | 0.871 |  | 0.001 | 0.991 |
| D2 | 0.000 | 0.000 | 0.001* | 0.000 | 0.000 | 0.001 | 0.000 | 0.000 | 0.003 | 0.001 |  | 0.000 |
| D3 | 0.003 | 0.001 | 0.001* | 0.209 | 0.000 | 0.998 | 0.996 | 0.001 | 0.509 | 0.991 | 0.000 |  |

Posthoc test using Games-Howell test, except (*) using Mann-Whitney test

**Supplementary material 6.** Descriptive Data, Normality Test, and Different Test of XTT Assay in 12 groups

| **Group** | **LDH Activity** | | | | | | | |
| --- | --- | --- | --- | --- | --- | --- | --- | --- |
|  | **Mean** | **Median** | **SD** | **Min.** | **Maks.** | **p*** | **p**** | **p***** |
| A1 | 63.69 | **50.09** | 18.07 | 48.36 | 86.07 | 0.009 | <0.001 | <0.001 |
| A2 | 45.07 | **43.38** | 5.71 | 41.97 | 57.92 | <0.001 |  |  |
| A3 | 41.69 | 42.57 | 5.07 | 31.48 | 47.75 | 0.171 |  |  |
| B1 | 35.95 | 34.31 | 5.42 | 28.93 | 43.31 | 0.551 |  |  |
| B2 | 29.73 | 27.22 | 8.73 | 19.68 | 42.29 | 0.427 |  |  |
| B3 | 47.80 | 51.51 | 11.51 | 25.54 | 60.72 | 0.338 |  |  |
| C1 | 70.44 | **55.47** | 19.88 | 53.57 | 95.06 | 0.009 |  |  |
| C2 | 57.28 | **55.16** | 7.14 | 53.41 | 73.34 | <0.001 |  |  |
| C3 | 46.24 | 47.21 | 5.57 | 35.01 | 52.90 | 0.171 |  |  |
| D1 | 45.88 | 43.83 | 6.78 | 37.10 | 55.08 | 0.550 |  |  |
| D2 | 10.51 | 9.46 | 7.62 | 2.15 | 25.85 | 0.184 |  |  |
| D3 | 12.35 | 11.17 | 3.06 | 9.55 | 18.01 | 0.223 |  |  |

p* = Shapiro-Wilk test; p** = Anova test ; p*** = Kruskal Wallis test

**Supplementary material 7.** Post Hoc Test of XTT Assay in 12 Treatment Groups

| **Group** | **A1** | **A2** | **A3** | **B1** | **B2** | **B3** | **C1** | **C2** | **C3** | **D1** | **D2** | **D3** |
| --- | --- | --- | --- | --- | --- | --- | --- | --- | --- | --- | --- | --- |
| A1 |  | 0.009* | 0.002* | 0.002* | 0.002* | 0.277* | 0.110* | 0.655* | 0.018* | 0.064* | 0.002* | 0.002* |
| A2 | 0.009* |  | 0.482* | 0.006* | 0.004* | 0.225* | 0.009* | 0.018* | 0.142* | 0.848* | 0.002* | 0.002* |
| A3 | 0.002* | 0.482* |  | 0.662 | 0.200 | 0.960 | 0.002* | 0.002* | 0.881 | 0.961 | 0.000 | 0.000 |
| B1 | 0.002* | 0.006* | 0.662 |  | 0.875 | 0.455 | 0.002* | 0.002* | 0.104 | 0.214 | 0.001 | 0.000 |
| B2 | 0.002* | 0.004* | 0.200 | 0.875 |  | 0.145 | 0.002* | 0.002* | 0.043 | 0.063 | 0.027 | 0.028 |
| B3 | 0.277* | 0.225* | 0.960 | 0.455 | 0.145 |  | 0.025* | 0.085* | 1.000 | 1.000 | 0.001 | 0.003 |
| C1 | 0.110* | 0.009* | 0.002* | 0.002* | 0.002* | 0.025* |  | 0.338* | 0.002* | 0.006* | 0.002* | 0.002* |
| C2 | 0.655* | 0.018* | 0.002* | 0.002* | 0.002* | 0.085* | 0.338* |  | 0.002* | 0.006* | 0.002* | 0.002* |
| C3 | 0.018* | 0.142* | 0.881 | 0.104 | 0.043 | 1.000 | 0.002* | 0.002* |  | 1.000 | 0.000 | 0.000 |
| D1 | 0.064* | 0.848* | 0.961 | 0.214 | 0.063 | 1.000 | 0.006* | 0.006* | 1.000 |  | 0.000 | 0.000 |
| D2 | 0.002* | 0.002* | 0.000 | 0.001 | 0.027 | 0.001 | 0.002* | 0.002* | 0.000 | 0.000 |  | 1.000 |
| D3 | 0.002* | 0.002* | 0.000 | 0.000 | 0.028 | 0.003 | 0.002* | 0.002* | 0.000 | 0.000 | 1.000 |  |

Posthoc test using Games-Howell test, except (*) using Mann-Whitney test
